# Characterisation of *Drosophila Ubx* ^*CPTI000601*^ and *hth* ^*CPTI000378*^ Protein Trap Lines

**DOI:** 10.1155/2014/191535

**Published:** 2014-10-15

**Authors:** Siew Woh Choo, Ching Yew Beh, Steven Russell, Robert White

**Affiliations:** ^1^Department of Genetics, University of Cambridge, Downing Street, Cambridge CB2 3EH, UK; ^2^Department of Oral Biology and Biomedical Sciences, Faculty of Dentistry, University of Malaya, 50603 Kuala Lumpur, Malaysia; ^3^Department of Physiology, Development and Neuroscience, University of Cambridge, Downing Street, Cambridge CB2 3DY, UK; ^4^Cambridge Systems Biology Centre, University of Cambridge, Downing Street, Cambridge CB2 EH, UK

## Abstract

In *Drosophila*, protein trap strategies provide powerful approaches for the generation of tagged proteins expressed under endogenous control. Here, we describe expression and functional analysis to evaluate new *Ubx* and *hth* protein trap lines generated by the Cambridge Protein Trap project. Both protein traps exhibit spatial and temporal expression patterns consistent with the reported endogenous pattern in the embryo. In imaginal discs, Ubx-YFP is expressed throughout the haltere and 3rd leg imaginal discs, while Hth-YFP is expressed in the proximal regions of haltere and wing discs but not in the pouch region. The *Ubx* ^*CPTI000601*^ line is semilethal as a homozygote. No T3/A1 to T2 transformations were observed in the embryonic cuticle or the developing midgut. The homozygous survivors, however, exhibit a weak haltere phenotype with a few wing-like marginal bristles on the haltere capitellum. Although *hth* ^*CPTI000378*^ is completely lethal as a homozygote, the *hth* ^*CPTI000378*^/*hth* ^*C1*^ genotype is viable. Using a hth deletion *(Df(3R)BSC479)* we show that *hth* ^*CPTI000378*^/*Df(3R)BSC479* adults are phenotypically normal. No transformations were observed in *hth* ^*CPTI000378*^, *hth* ^*CPTI000378*^/*hth* ^*C1*^, or *hth* ^*CPTI000378*^/*Df(3R)BSC479* embryonic cuticles. We have successfully characterised the Ubx-YFP and Hth-YFP protein trap lines demonstrating that the tagged proteins show appropriate expression patterns and produce at least partially functional proteins.

## 1. Introduction

In* Drosophila*, enhancer trap strategies allow rapid access to expression patterns, molecular data, and mutations in trapped genes. However, they do not give any information at the protein level, for example, about the subcellular localization of proteins. The ectopic expression of tagged proteins, in particular fusions with fluorescent tags such as the green fluorescent protein (GFP) and its derivatives, allows a dynamic study of fusion protein behaviour in unfixed, living cells and tissues. In addition, epitope-tagged proteins, carrying tags such as FLAG or Myc, can be generated by inserting an oligonucleotide sequence encoding the tag into a gene of interest and subsequently expressing the hybrid protein* in vivo*. This technique has been successfully employed in yeast where pioneering studies [1, 2] have tagged all 203 different transcription factors with a Myc epitope tag. The tag sequence was introduced into the C-terminus of each transcription factor* in situ* by homologous recombination. However, there are difficulties associated with this technique in organisms where homologous recombination is not efficient.

One way of overcoming some of these limitations is via the use of protein traps [3, 4]. In this approach, a vector carrying a tag exon flanked by splice acceptor (SA) and donor sites (SD) is randomly inserted into the genome via transposable elements. If the transposon is inserted into an intron of an endogenous gene in the correct frame and orientation, a tag-expressing fusion protein may be generated [5]. Since these fusion proteins are expressed from the host gene's native regulatory elements, the proteins should show similar spatial and temporal expression patterns as the endogenous gene. In* Drosophila*, this approach has been successfully tested by Morin et al. [4] who inserted a GFP exon into the* Drosophila* genome using P-elements, successfully generating a large number of protein trap lines. Subsequently, larger scale studies have generated many more protein trap insertions [6, 7].

One advantage of this approach is that it can generate a collection of protein traps with the same tag, for example, GFP; therefore, one can, for example, study DNA-protein interactions for a large number of transcription factors in a systematic way without requiring many different antibodies. Chromatin immunoprecipitation (ChIP) is one such powerful technique for studying protein-DNA interactions in cells, and there are many possible ways to perform such experiments [10–12]. Using tagged proteins enables systematic ChIP using a well-characterised tag-specific antibody. However, before using the protein trap lines for further applications, it is important to establish that the proteins traps are expressed in the correct spatial and temporal pattern reflecting the expression of the endogenous genes. In the present study, we describe an expression and functional analysis evaluating protein trap insertions in both* Ubx* and* hth* genes.

## 2. Materials and Methods

### 2.1. Fly Lines and Antibodies

#### 2.1.1. Protein Trap Lines

The transgenic Ubx-YFP (*Ubx* ^*CPTI000601*^) and Hth-YFP (*hth* ^*CPTI00378*^) FlyProt protein trap lines were generated via a PiggyBac transposon-based exon-trapping screen [13]. For each protein trap line, an exon carrying a yellow fluorescent protein (YFP) marker along with affinity purification epitopes was introduced into the endogenous gene. Wild type flies were the* w* ^*1118*^ line used to generate the protein traps. Flies were maintained on standard cornmeal-yeast agar at 25°C or 18°C.

#### 2.1.2. Antibodies

Table 1 is a summary of the antibodies used in this study.

### 2.2. Examination of Protein Trap Phenotype

#### 2.2.1. Lethality Assay

Heterozygous* Ubx* ^*CPTI000601*^ or* hth* ^*CPTI000378*^ flies were crossed in vials and kept at 25°C for two days and the adults removed. The number of heterozygous and homozygous flies enclosing was scored each day for each vial.

#### 2.2.2. Cuticle Preparations

Embryos aged 18–24 hours after egg laying (AEL) were collected on apple-juice-agar plates from a cage held at 25°C. Embryos were dechorionated with commercial bleach for 3 min and rinsed with water. Embryos were transferred to a small glass vial containing 1 : 1 n-heptane : methanol (BDH, Analar grade) and shaken vigorously for 10–15 seconds. Devitellinised embryos were transferred to a clean tube and washed twice in methanol. To mount preparations, embryos were transferred to a clean microscope slide and a few drops of Hoyer's lactic acid 1 : 1 were added. A coverslip was gently placed on the sample. Embryos were incubated in Hoyer's medium at 65°C overnight. The cuticle preparations were examined by dark field microscopy.

#### 2.2.3. Midgut Analysis

Embryos aged 18–24 hours AEL were collected on apple-juice-agar plates from a cage held at 25°C. Embryos were dechorionated with commercial bleach for 3 min and rinsed with water. The embryos were transferred to a clean microscope slide and mounted in Citifluor (VWR) under a coverslip. The midgut morphology was examined using a standard Zeiss Axiophot microscope (Filter: BP 546; FT 580; LP 590).

### 2.3. Histology

#### 2.3.1. Preparation and Immunostaining of Embryos

Embryos aged 0–16 hours AEL were collected from the* Ubx* ^*CPTI000601*^ or* hth* ^*CPTI000378*^ lines at 25°C. Embryos were washed with tap water and dechorionated in a solution of commercial bleach at room temperature (RT). Embryos were washed with water and fixed with 4% formaldehyde for 30 minutes at RT. Fixed embryos were washed twice in PTX (PTX; PBS, 0.1% Triton X-100) and once with PBTX (PBTX; PBS, 0.1% BSA, 0.1% Triton X-100). After washing, embryos were incubated in PBTX rolling for 2 hours at 4°C to block nonspecific protein binding sites. PBTX was replaced with a primary *α*-GFP antibody (Molecular Probes) diluted in PBTX and incubated overnight at 4°C. The primary antibody was removed and the embryos were washed 3 times with PBTX and incubated for 1 hour at 4°C with rolling. Alexa 488 labelled secondary antibody (Molecular Probes) was added and incubated for 1 hour 30 minutes at RT. The embryos were then washed 3 times with PBTX over a 1-hour period at RT. After removing the excess PBTX, the embryos were mounted in Citifluor and visualized using a Zeiss Axiophot fluorescence microscope.

To double-label the embryos, the same procedure as described above was performed except with the use of two primary antibodies followed by subsequent incubation with two species-specific secondary antibodies.

#### 2.3.2. Preparation and Double-Labelling of Imaginal Discs

Larval tissues were dissected in PBS and fixed in 4% formaldehyde for 20 minutes. The fixed larval heads were rinsed 3X in PBX (PBX; PBS + 0.2% Triton X100). The tissues were blocked in 0.1% BBX (BBX; PBX, 0.1% BSA) for 30 minutes. Two primary antibodies were added in 0.1% BBX in a total volume of 50 *μ*L and incubated overnight at 4°C. After incubation with the primary antibodies, 0.1% BBX was removed and the tissues were washed 3 times in PBX (15 minutes for each wash). A total volume of 50 *μ*L of the secondary antibodies prepared in 0.5% BBX was added and incubated for 1 hour and 30 minutes at RT. Tissues were then washed 3 times in PBX (15 minutes for each wash) and were fixed with 4% formaldehyde for 20 minutes at RT. The tissues were rinsed 3 times (5 minutes for each wash) in PBX and left in Citifluor overnight at 4°C. The imaginal discs were dissected and mounted in Citifluor and viewed with a spinning disc confocal microscope (see below).

### 2.4. Microscopy and Software

For imaging of embryos, a Zeiss Axiophot fluorescence microscope with an attached QImaging camera was used. These images were recorded with QCapture Pro version 5.1.1.14 software and processed in Photoshop CS (Adobe). For the imaging of imaginal discs, Yokogawa CSU10 spinning disc confocal microscopy with a Nikon eclipse E1000 microscope and a Hamamatsu Electron Multiplier CCD Digital Camera C9100-13 was used. These images were recorded with Volocity version 4.3.2 software (Improvision) and processed in Photoshop CS (Adobe).

## 3. Results

In this study, we took advantage of two protein trap lines generated by the Cambridge Protein Trap project [13] using a PiggyBac transposable element to randomly insert a YFP exon into the* Drosophila* genome (Supplementary Data available online at http://dx.doi.org/10.1155/2014/191535).* Ubx* ^*CPTI000601*^ and* hth* ^*CPTI000378*^ are YFP protein trap insertions in* Ubx* and* hth,* respectively. In the case of* Ubx*, the YFP exon is inserted into the last intron of the gene at genomic position chr3R:12486327. The inserted exon is in the same frame as all six known alternatively spliced transcript variants of* Ubx*. The* hth* ^*CPTI000378*^ line is an insertion at genomic position chr3R:6381126 in the endogenous* hth* gene. The insertion traps all but the two shortest* hth *spliced transcript variants (*hth*-RE and* hth*-RF).

To examine the protein trap expression pattern and confirm the suitability of lines for further applications, we first determined their expression patterns by immunolabelling 0–16 h* Drosophila* embryos with rabbit *α*-GFP/YFP antibodies [14] and visualising the stained embryos using fluorescent microscopy. The observed expression patterns were compared with the endogenous expression patterns as reported in the published literature. We chose to use immunohistochemistry rather than direct observation of YFP fluorescence from the protein trap line because it provides better sensitivity for examining the expression patterns, especially with the Hth-YFP line in embryos.

### 3.1. The Spatial and Temporal Expression Patterns of Ubx-YFP Line

**Figure 1:**
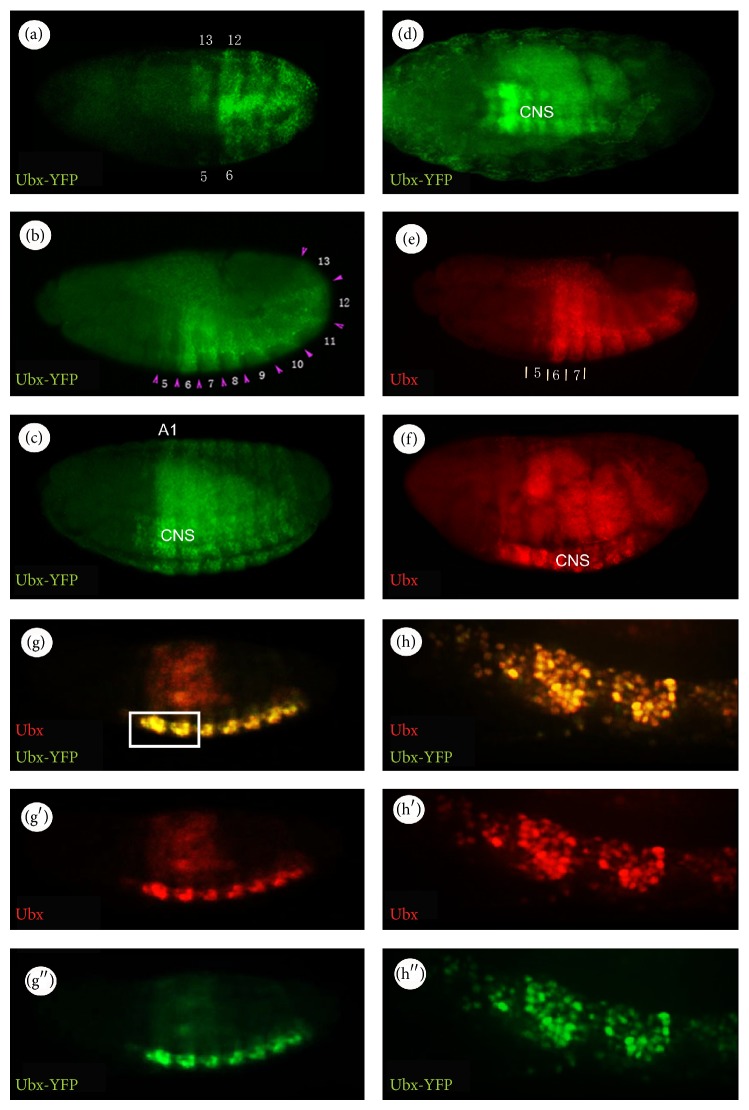
Ubx-YFP expression patterns during embryogenesis. ((a)–(d)) Ubx-YFP labelling; (a) Stage 10 embryo: Ubx-YFP expression is detected in PS5–13. The indicated numbers refer to parasegments; (b) Stage 12-13 embryo: Ubx-YFP is expressed in the ectoderm in PS5–13. Ubx-YFP shows heterogeneous patterns of expression both within and between the parasegments; arrowheads mark parasegment boundaries; (c) Stage 15; (d) Stage 16. ((e)-(f)) Similar expression is observed for the endogenous* Ubx* gene detected by immunostaining in wild type embryos using *α*-Ubx antibody; (e) Stages 12-13; (f) Stage 15. ((g)–(g′′)) Double-labelling of Ubx-YFP and its endogenous gene using *α*-GFP and *α*-Ubx antibodies, respectively, in* Ubx* ^*CPTI000601*^ embryos. ((h)–(h′′)) a high magnification of a region boxed in (g). Double-labelling shows that the protein trap has very similar temporal and spatial expression patterns to its endogenous gene. All embryos are viewed laterally with anterior to the left.

The* Ubx* expression pattern has been well characterized in a number of studies [8, 15]. Briefly, in wild type embryos,* Ubx* expression is first detected around cellularisation at approximately 3 hours AEL. The expression then becomes clearly defined by Stage 10 of embryogenesis and is more prominent in the central nervous system (CNS) and ectoderm at later stages. One of the unique characteristics of* Ubx* expression is its metameric pattern with expression from parasegments (PS) 5 to 13. In line with the endogenous pattern, Ubx-YFP is significantly detected from approximately Stage 10 of embryogenesis (Figure 1(a)) and thereafter becomes prominent in the CNS and ectoderm after germ band extension. Ubx-YFP expression is observed in a restricted region in PS5–13 (Figures 1(b) and 1(c)). At Stages 15–16 of embryogenesis, the fluorescent signal becomes very strong in the CNS (Figure 1(d)).

**Figure 2:**
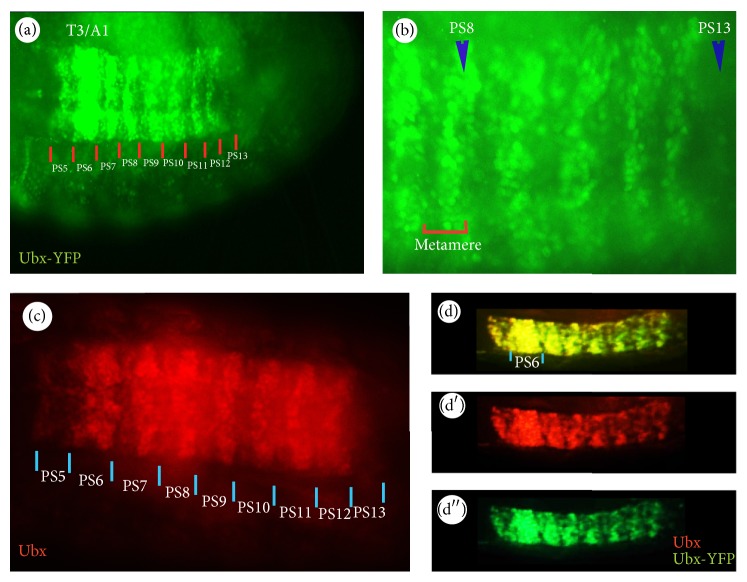
Metameric and heterogeneous pattern of* Ubx* expression in the CNS. (a) Ubx-YFP expression pattern in the Stage 16 embryo as detected with *α*-GFP in* Ubx* ^*CPTI000601*^. Labelling is very prominent in PS6. (b) A magnified view of a part of the embryo shown in (a) demonstrating nuclear staining in metameres with PS8 and 13 highlighted. Both (a) and (b) are ventral views with anterior to the left. (c) Endogenous* Ubx* expression pattern as revealed by staining the wild type with mouse *α*-Ubx antibody. ((d)–(d′′)) Double staining of Ubx-YFP and its endogenous gene using *α*-GFP and *α*-Ubx antibodies, respectively, in the CNS of* Ubx* ^*CPTI000601*^ embryos. The protein trap and overall Ubx labelling have very similar temporal and spatial expression patterns.

As described before, a characteristic feature of Ubx distribution in the CNS is its metameric pattern [17]. For example, as shown in Figure 2, Ubx-YFP is expressed in a series of repeat units, called metameres. The boundaries of the metamere do not coincide with the boundaries of the segmental neuromere and epidermis [17, 18], but they coincide with the parasegmental boundaries. In Figure 2, Ubx-YFP is expressed in parasegments PS5–PS13, with the most prominent labelling in PS6, which spans the 3rd thoracic (T3) and the 1st abdominal segments (A1). The intensity of the fluorescent signal declines posteriorly and becomes very weak by PS13.

Another interesting feature of Ubx expression is its heterogeneity both within and between parasegments (Figures 1(b), 1(e), and 2). All these features are reflected in the Ubx-YFP expression. Ubx-YFP expression declines when moving from T3 to A8 (or PS5–PS13) because* Ubx* is repressed by the more posteriorly expressed homeotic genes* abd-A* and* Abd-B* [19]. Such heterogeneity in* Ubx* expression can be also seen within a metamere (Figure 2(b)). For example, the labelling is strong in the posterior part of a metamere compared with the anterior portion. The heterogeneity is most extreme in PS13 where only a few nuclei are labelled in the posterior region of the metamere.

**Figure 3:**
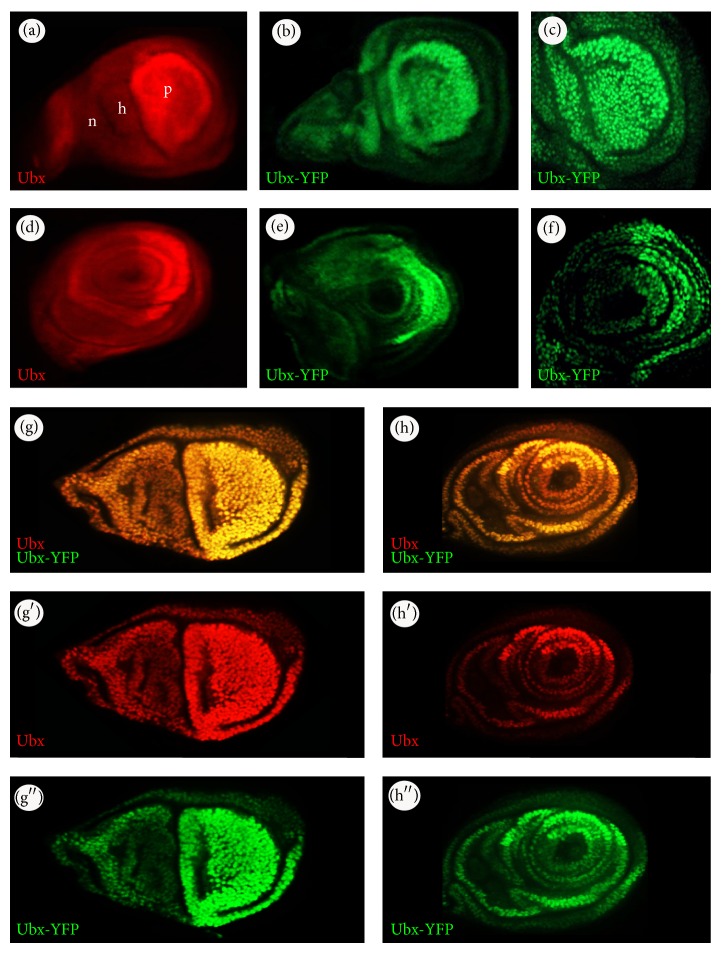
The patterns of Ubx expression in the T3 haltere and leg discs. (a) The expression patterns of endogenous Ubx in T3 haltere: p = pouch, h = hinge, and n = notum. (b) The expression pattern of Ubx-YFP in haltere and (c) at higher magnification. (d) The expression patterns of the endogenous Ubx in T3 leg disc. (e) The expression pattern of Ubx-YFP in T3 leg disc and (f) at higher magnification. The expression pattern of Ubx-YFP at high magnification shows that the protein trap is nuclear. Double staining of the Ubx-YFP and its endogenous protein using *α*-GFP and *α*-Ubx, respectively, in the* Ubx* ^*CPTI000601*^ haltere ((g)–(g′′)) and T3 leg ((h)–(h′′)) imaginal discs shows that they have very similar temporal and spatial expression patterns.

In wild type T2/T3 imaginal discs,* Ubx* is expressed throughout the T3 haltere and T3 leg imaginal discs of third instar larvae (Figures 3(a) and 3(d)), but it shows little expression in the T2 wing imaginal disc. In the haltere, the expression of* Ubx* is very strong in the pouch region. In the T3 leg disc,* Ubx* is expressed strongly in the posterior half of the T3 leg disc and is weaker in the anterior half (Figure 3(d)). We directly visualized Ubx-YFP expression patterns in these discs using confocal microscopy and found that, as in wild type, the protein trap is expressed in nuclei throughout both T3 imaginal discs (Figure 3). In line with the endogeneous Ubx pattern, Ubx-YFP is restricted to peripodial membrane nuclei in the T2 wing imaginal disc (data not shown). Double staining of the Ubx-YFP protein trap and its endogenous gene in* Ubx* ^*CPTI000601*^ imaginal discs and embryos shows that they have similar temporal and spatial expression patterns (Figures 1–3). Importantly, we do not observe any YFP expressing cells that do not stain with the Ubx antibody or vice versa.

Taken together, we conclude that the* Ubx* protein trap shows a spatial and temporal expression pattern consistent with the previously reported endogenous pattern in both the embryo and imaginal discs.

### 3.2. The Spatial and Temporal Patterns of Hth-YFP Expression

**Figure 4:**
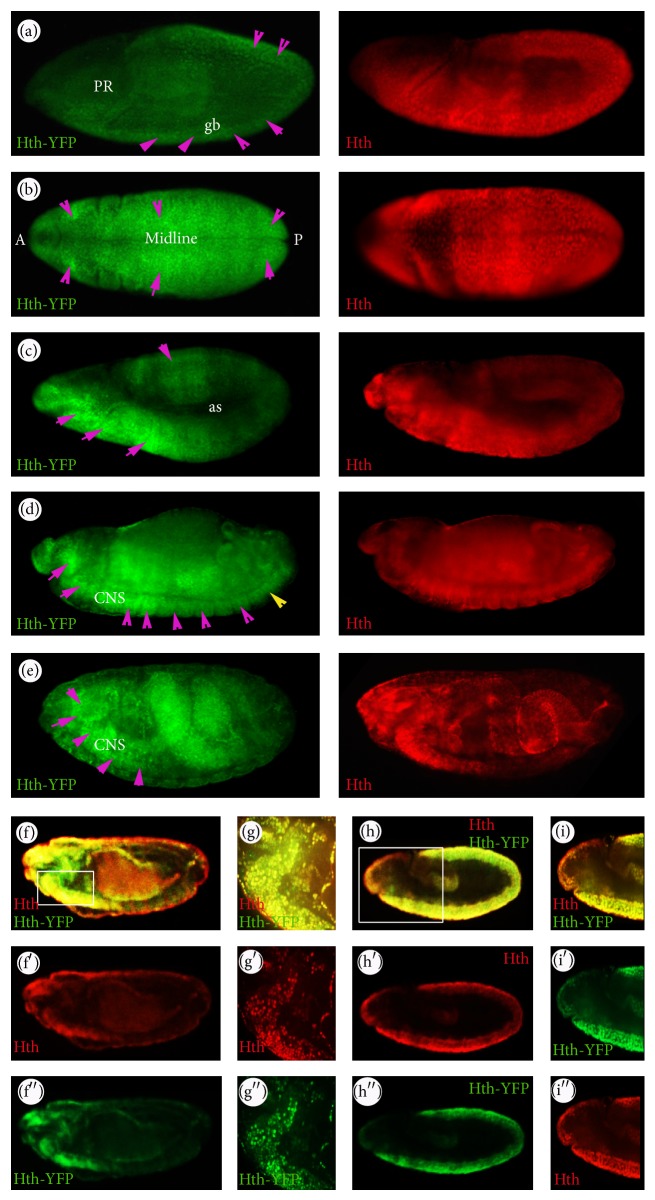
Patterns of Hth-YFP expression. ((a)–(e)) Left panels: Hth-YFP embryos, stained with *α*-GFP antibodies; Hth-YFP expression is indicated by purple arrowheads. Right panels: the distribution of the endogenous Hth protein for the corresponding embryonic stages in the wild type. (a) Lateral view of a Stage 9 embryo, PR: procephalon, gb: germ band. (b) Dorsal view of a Stage 10 embryo. A: anterior and P: posterior. (c) Stage 11 embryo; Hth-YFP is strongly expressed anteriorly but declines posteriorly. (d) Stage 13 embryo. The posterior extent of the CNS is indicated by the yellow arrow. (e) Late stage of embryogenesis. Hth-YFP expression is prominent in the CNS of the Stage 15 and Stage 16 embryos but becomes weaker in the posterior regions. ((f)–(i′′)) Hth-YFP embryos stained with *α*-GFP and *α*-Hth antibodies. ((g)–(g′′)) High magnification of a region boxed in (f), whereas ((i)–(i′′)) are high magnifications of a region boxed in (h). Overall, the protein trap shows similar temporal and spatial expression patterns to the endogenous Hth protein in the embryo.

Endogenous* hth *expression in embryos was characterized in a previous study [20]. In wild type embryos,* hth *is expressed broadly throughout the embryo but not in the procephalon (Figure 4). The expression becomes stronger in anterior regions but declines in the posterior region during later stages of embryogenesis (from Stage 10 onwards). In line with the endogenous pattern, Hth-YFP shows widespread expression in the* Drosophila* embryo throughout embryogenesis (Figure 4). At Stage 9, Hth is expressed throughout the embryo, except for the procephalon (Figure 4(a)), and the protein trap is similarly expressed although the labelling is weak. From Stage 10, the labelling is very prominent in the nuclei of ectodermal cells. From Stage 11, the expression of Hth-YFP is strong in the head thoracic segments and declines in intensity in the abdominal segments. This expression continues and, by late embryogenesis, Stages 15–16, Hth-YFP expression is more evident in the nuclei of neuronal cells and the expression in the abdominal segments declines further. Hth-YFP also shows a strong anterior-to-posterior expression gradient along the CNS. All of these features are in line with the endogenous* hth *expression in the embryo (Figure 4).

**Figure 5:**
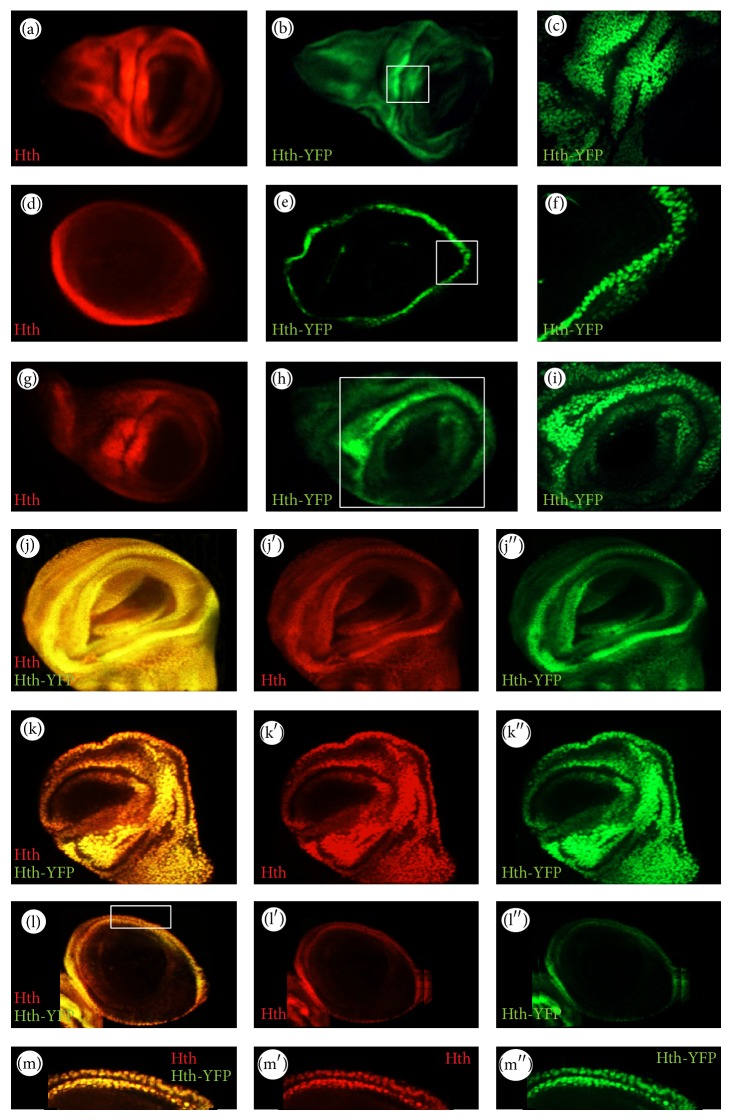
The patterns of Hth expression in T2 and T3 imaginal discs. ((a)–(c)), ((d)–(f)), and ((g)–(i)) are T2 wing, T3 leg, and T3 haltere discs, respectively. ((a), (d), and (g)) The expression patterns of endogenous Hth; ((b), (e), and (h)) the expression patterns of Hth-YFP; ((c), (f), and (i)) the expression patterns of Hth-YFP in the boxed regions in the corresponding images at high magnification showing that the protein trap is nuclear. Double-labelling of Hth-YFP and its endogenous gene shows that they have very similar temporal and spatial expression patterns in wing ((j)–(j′′)), haltere ((k)–(k′′)), and T3 leg ((l)–(l′′)) imaginal discs. ((m)–(m′′)) are higher magnifications of the boxed region in (l).

In the wild type third larval instar haltere and wing discs, Hth is expressed everywhere except the pouch (Figures 5(a)–5(c) and 5(g)–5(i)). As shown in the figure, Hth-YFP has similar patterns to the endogenous gene and it is nuclear. Furthermore, Hth-YFP is expressed in the periphery of the T3 leg disc (Figures 5(d)–5(f)), similar to the pattern of the endogenous protein. Double staining of the Hth-YFP protein trap and the endogenous protein in* hth* ^*CPTI000378*^ imaginal discs (as well as embryos) also shows that they have similar temporal and spatial expression patterns (Figures 4 and 5). Taken together, as with the* Ubx* protein trap, we conclude that the* hth *protein trap is also expressed in line with the expression of the endogenous gene.

### 3.3. Functional Analysis of Protein Trap Lines

Although the preceding analysis indicates that the expression of the protein trap lines mirrors endogenous protein expression, it is possible that these fusion proteins may not provide the same functions as the endogenous proteins. To assess this, we performed functional characterisations of the* Ubx* ^*CPTI000601*^ and* hth* ^*CPTI000378*^ lines.

#### 3.3.1. *Ubx* ^*CPTI000601*^ Protein Trap

*Survival Analysis*. The* Ubx* ^*CPTI000601*^ line is semilethal as a homozygote, with some flies surviving to adulthood. To define the degree of lethality, we crossed heterozygous* Ubx* ^*CPTI000601*^ flies and scored the F1 generation (Table 2). Of 525 adult flies, 54 are homozygous and the remainder heterozygous. Since we expect 1/3 (175 flies) of the F1 to be homozygous, we conclude that 31% of homozygous* Ubx* ^*CPTI000601*^ flies survive. With* Ubx* null mutations such as* Ubx* ^*1*^, escapers are never observed. This supports the view that the CPT-000601 insertion can provide partial* Ubx* function and is not null.

**Figure 6:**
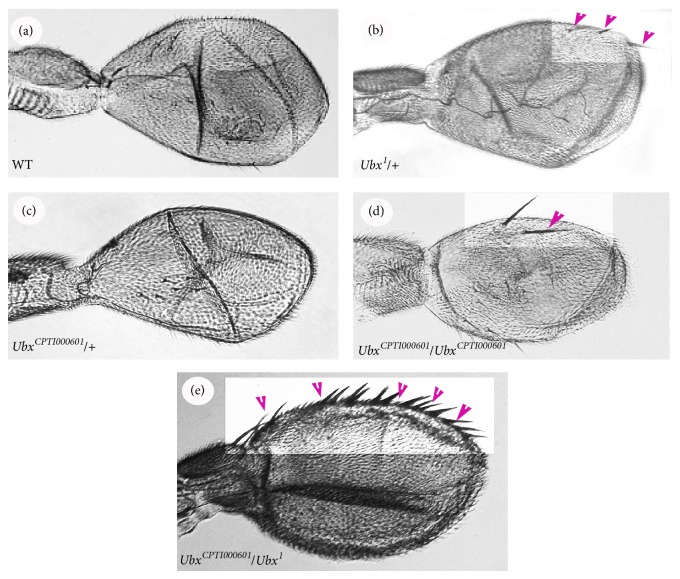
Comparison between wild type* Ubx* ^*CPTI000601*^ and* Ubx* ^*1*^ halteres. (a) Wild type haltere. (b)* Ubx* ^*1*^/+ haltere. Several marginal bristles are observed. (c)* Ubx* ^*CPTI000601*^/+ haltere. (d)* Ubx* ^*CPTI000601*^/*Ubx* ^*CPTI000601*^ haltere. (e)* Ubx* ^*CPTI000601*^/*Ubx* ^*1*^ haltere. Wing-like marginal bristles are observed. Purple arrowheads indicate bristles.

*Analysis of Haltere Phenotype*.* Ubx* mutations generally show homeotic transformations in the larval cuticle and in genotypes that survive to adulthood, in the halteres. For example, with the amorphic* Ubx* ^*1*^ allele, we can see haltere-to-wing transformation in heterozygotes (Figure 6(a)). The haltere-to-wing transformation is generally characterised by wing-type bristles along the anterior margin of the haltere. As shown in Figure 6(b), in* Ubx* ^*1*^/+ halteres, a few wing-type marginal bristles (3 ± 1.4 bristles; 33 halteres) are observed. We dissected* Ubx* ^*CPTI000601*^ halteres from each genotype and examined them via light microscopy. In the case of heterozygous* Ubx* ^*CPTI000601*^/+ halteres, no wing-type marginal bristles were observed (Figure 6(c)). In contrast, with homozygous* Ubx* ^*CPTI000601*^ halteres, a few wing-type bristles (2.4 ± 1.2 bristles; 25 halteres) were observed on the haltere capitellum (Figure 6(d)). If we consider that* Ubx* ^*1*^/+ flies have approximately 50% normal* Ubx* function, then it is reasonable to assume that the* Ubx* ^*CPTI000601*^ flies have at least 50% of normal* Ubx* function. In addition, we also examined heterozygous* Ubx* ^*CPTI000601*^/*Ubx* ^*1*^ halteres and found that the anterior margin bristles (23.6 ± 4.6 bristles; 18 halteres) were crowded compared to* Ubx* ^*1*^/+ halteres (Figure 6(e)). The much stronger phenotype observed when the* Ubx* ^*1*^ null allele is in trans with* Ubx* ^*CPTI000601*^ supports the view that the protein trap allele has reduced* Ubx* function and is thus a weak hypomorph.

**Figure 7:**
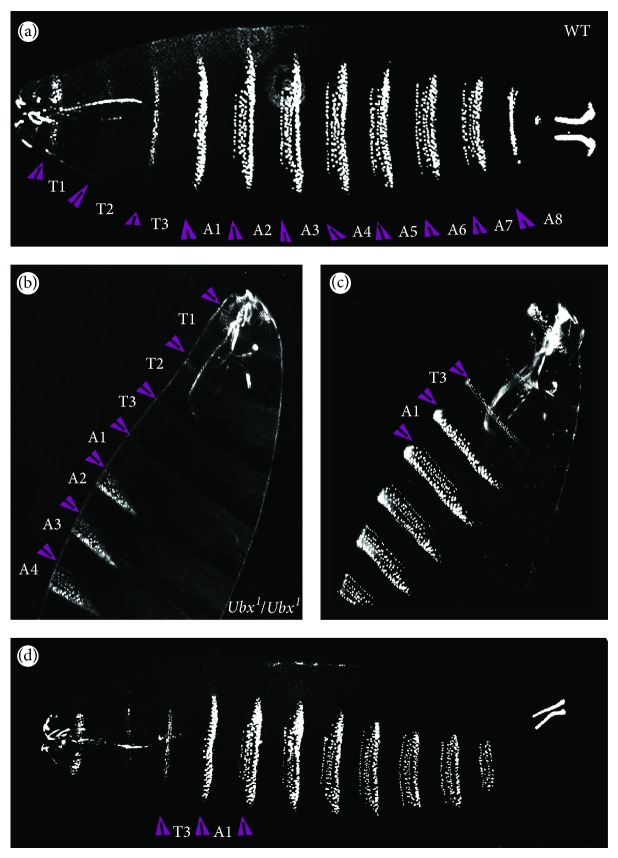
Cuticle patterns of embryos carrying Ubx-YFP protein trap. (a) Wild type embryo. (b) A diagram showing that both T3 and A1 segments are transformed into T2 in a* Ubx* loss of function mutant. Both transformed segments show T2-like denticle patterns. ((c) and (d)) Different views of Ubx-YFP embryos. Denticle bands of T3 and A1 segments are normal in Ubx-YFP embryos. Purple arrowheads indicate the parasegments.

*Cuticle Analysis*. In* Ubx* loss of function mutants such as* Ubx* ^*1*^, the T3 and A1 segments are transformed to T2 segments [16] and this phenotype can be observed in the denticle patterns of the larval cuticles in (Figure 7(b)). To further characterise the* Ubx* ^*CPTI000601*^ phenotype, we examined cuticle preparations from a* Ubx* ^*CPTI000601*^ stock to check for T3/A1-to-T2 transformations. We examined the denticle patterns of* Ubx* ^*CPTI000601*^ embryos collected between 16 and 24 hours AEL. In the wild type, the three thoracic segments (T1, T2, and T3) have a characteristic thinner denticle pattern compared to the denticles on the abdominal segments; transformation of posterior to anterior fates (e.g., A1-T2 transformation) is readily observed as thinner denticle bands in the A1 segment. However, out of 230 cuticles examined, no transformations were observed (Figure 7), again supporting the view that* Ubx* ^*CPTI000601*^ can provide sufficient* Ubx* function for grossly normal embryonic development.

**Figure 8:**
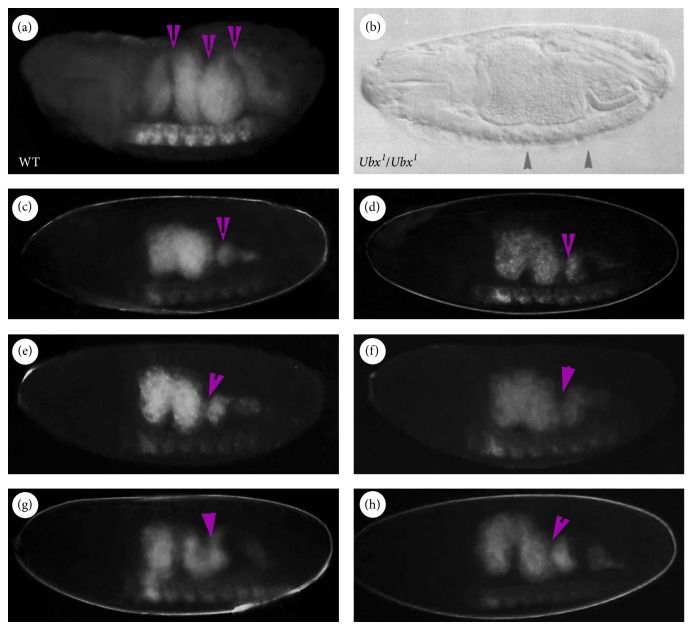
Midgut analysis. (a) Wild type midgut with all 3 constrictions indicated by purple arrowheads. Left arrowhead: 1st constriction; middle arrowhead: 2nd constriction; right arrowhead: 3rd constriction. (b) The 2nd constriction is missing in homozygous* Ubx* ^*1*^ mutant embryos [16]. (c)–(h) show several examples of Ubx-YFP embryos with complete midguts. The 2nd constriction of the midgut is indicated by purple arrowhead. Panel B is reproduced from [16] with permission.

*Midgut Analysis*. In homozygous* Ubx* ^*1*^ mutant embryos, the second constriction of the embryonic midgut is missing [16] and to assess the protein trap we examined the midgut of ~35* Ubx* ^*CPTI000601*^ embryos at Stages 15–16 by fluorescence microscopy. We found that the midgut in all of these embryos had a wild type set of constrictions, providing further evidence that the protein trap line has substantial Ubx function (Figure 8).

#### 3.3.2. *hth* ^*CPTI000378*^ Protein Trap

*Survival Analysis*. The* hth* ^*CPTI000378*^ protein trap line is lethal as a homozygote. To confirm the degree of lethality, we crossed heterozygous* hth* ^*CPTI000378*^ flies and scored the F1 generation using a similar procedure described above. Of 857 adult flies, all are heterozygous, suggesting that this line is completely lethal as a homozygote.

To determine whether* hth* ^*CPTI000378*^ is a null allele, we crossed the* hth* ^*CPTI000378*^ */TM6C* with* hth* ^*C1*^/*TM2* flies:* hth* ^*C1*^ is a strong hypomorphic allele [21]. The crosses generate progeny with genotypes* hth* ^*C1*^/*TM6C*,* hth* ^*C1*^/*hth* ^*CPTI000378*^,* TM2*/*TM6C,* and* hth* ^*CPTI000378*^/*TM2*.  Table 3 summarises the number of observed progeny for each genotype. Encouragingly, we observed viable* hth* ^*C1*^/*hth* ^*CPTI000378*^ flies in expected Mendelian ratio (Table 3), suggesting that* hth* ^*CPTI000378*^ retains at least some* hth* functions.

Although we observed that* hth* ^*CPTI000378*^/*hth* ^*C1*^ flies are viable, it is possible that there was complementation between alleles. To further test this possibility, we crossed the heterozygous* hth* ^*CPTI000378*^ flies with* Df(3R)BSC479* flies, which carry a* hth *deletion. Again, we observed phenotypically normal* hth* ^*CPTI000378*^/*Df(3R)BSC479* adults, further supporting the view that the* hth* ^*CPTI000378*^ allele is at least partially functional. This suggests that the lethality associated with* hth* ^*CPTI000378*^ is due to a second site mutation and not to a lesion in* hth*.

*Cuticle Analysis*. In homozygous* hth* ^*C1*^ embryos, A1 shows an A5-like phenotype and the thoracic segments show an abdominal-like phenotype in the denticle patterns of the larval cuticles [21]. To characterise the* hth* ^*CPTI000378*^ phenotype, we examined cuticle preparations from a* hth* ^*CPTI000378*^ stock to check for posterior transformations. We examined the denticle patterns of* hth* ^*CPTI000378*^ embryos collected between 16 and 24 hours AEL with no transformations observed (*n* = 173). Moreover, no phenotypes were observed for* hth* ^*CPTI000378*^/*hth* ^*C1*^ in 209 cuticles examined. In addition, we also examined the cuticles of* hth* ^*CPTI000378*^/*Df(3R)BSC479* and saw no phenotypes in 178 cuticles. These results again indicate that* hth* ^*CPTI000378*^ can provide substantial* hth* functions. Taken together, the observations from the genetic crosses described above and the cuticle analyses suggest that the* hth* ^*CPTI000378*^ allele is not a null allele and support the view that the* hth* ^*CPTI000378*^ lethality is likely due to a second site mutation.

### 3.4. Double Ubx and Hth Labelling

**Figure 9:**
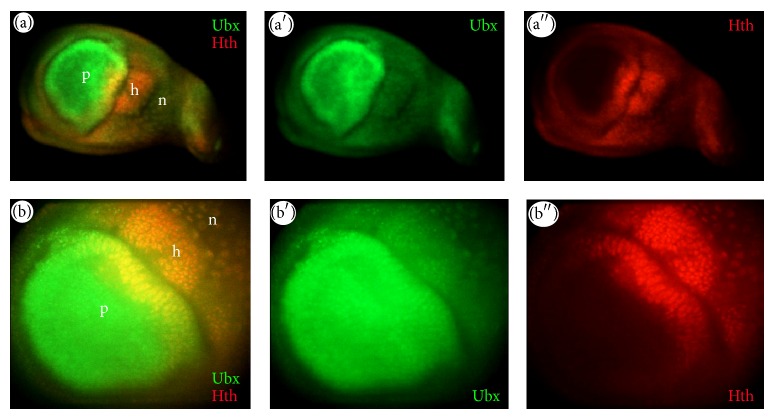
Double-labelling of Ubx and Hth in haltere discs. ((a)–(a′′)) Ubx and Hth are colocalised in hinge (h) and notum (n) regions but show different expression levels ((b)–(b′′)) of Ubx and Hth in a specific region of the dorsal hinge (h); p = pouch; n = notum; h = hinge.

One interesting observation when examining Ubx-YFP and Hth-YFP expression patterns in the haltere is that both protein traps are expressed in proximal scabellum and pedicel “hinge” regions (Figures 3 and 5). However, in the dorsal pedicel, Hth-YFP has a very high expression level compared to Ubx-YFP, which is very weak. This feature has not been reported in published literature. To confirm whether this feature is also seen in wild type discs, we performed double-labelling assays using *α*-Ubx and *α*-Hth in wild type discs. Double-labelling confirms that, while both transcription factors are coexpressed in the dorsal pedicel, in line with the protein trap expression, only Hth is specifically more strongly expressed in this region (Figure 9). This could be an interesting observation in light of the fact that* hth* has been shown to be required for the development of the analogous hinge region of the wing disc [22]. The high expression level of Hth may be critical for this transcription factor to specify proximal structures.

## 4. Discussion

In this study, we present several lines of evidence validating the expression and function of protein traps for the Hox protein Ubx and the Hox cofactor Hth. First, the two protein traps have expression patterns that are similar to their endogenous proteins in both embryos and specific imaginal tissues, as revealed either by immunostaining assays or by directly visualizing YFP expression. Although both protein trap lines mimic the endogenous patterns of expression, we also performed a functional check by examining the phenotypes associated with each line. Briefly, the* hth* ^*CPTI000378*^ line is homozygous and lethal; however, we recover viable and phenotypically normaladult flies when the protein trap line is hemizygous or in combination with a strong hypomorph. We therefore conclude that the CPTI-000378 protein trap can provide substantial normal function and that the chromosome carries a second site mutation.

In contrast, the* Ubx* ^*CPTI000601*^ insertion allele is semilethal as a homozygote with survivors exhibiting a weak haltere phenotype. Importantly, we show that there is no phenotype observed in the embryonic cuticle or in the developing midgut, two other regions which require* Ubx* function. These observations suggest that normal* Ubx* function might be slightly affected during haltere development but that the Ubx-YFP protein trap is sufficient for normal embryonic development.

A potential limitation of the* hth* ^*CPTI000378*^ line is that not all* hth *splice variants are trapped by the YFP exon, with the two shortest isoforms terminating before the trapped intron. Interestingly, all of the* hth* transcript variants trapped by the YFP exon encode proteins containing a homeodomain whereas the gene products of the two untrapped splicing isoforms lack the DNA binding domain and therefore may not directly bind to DNA. All known* Ubx* splicing variants are successfully trapped with the YFP exon in* Ubx* ^*CPTI000601*^, but this line is semilethal. An interesting question is why the line is semilethal. We cannot rule out the possibility that there is a difference between the relative levels of the tagged and wild type proteins for each isoform.

In previous studies,* hth* has been shown to act as a cofactor for HOX proteins [23–26]. In this study, we showed that the* hth* ^*CPTI000378*^ protein trap is broadly expressed in all HOX domains in the embryo, reinforcing its role as a HOX cofactor. Moreover,* hth* is also expressed in cells where Hox genes are not expressed; for example, we showed that the Hth-YFP protein trap is expressed in the hinge and notum regions (but not the pouch) of the wing disc where no* Hox* genes, including* Ubx*, are significantly expressed [27]. Consequently, it is likely that* hth* functions independently of Hox proteins in most of the cells in the wing disc. During the late stages of embryogenesis, the expression level of* hth* is very high in the anterior region, especially in the head, where HOX proteins are not expressed. The role of* hth* in head development has previously been studied [28–30]. For example,* hth* is not expressed in cells that give rise to the eye but ectopic expression of* hth* in these cells can block eye formation [31]. Moreover,* hth* is also required for the development of ventral head structures by preventing eye formation in this region. In addition,* hth* has also been shown to be involved in antennal development [28]; induction of* hth* mutant clones in the antennal region results in an antenna-to-leg transformation. Therefore, the high expression level of* hth* that we observed in the head region may indicate that* hth* plays an important role in head development.

## 5. Conclusion

The successful characterization of the two protein traps provides validated resources for studying the function of* Ubx* and* hth*. Not only do protein traps provide a transcriptional readout of the two genes, but also their protein localization patterns can be important for deciphering gene functions. Using these two protein traps, we mapped the binding sites of both transcription factors and identified their* in vivo* target genes in the embryo and specific imaginal discs [32]. Importantly, one of the advantages of trapping these transcription factors with YFP exon is that we can visualize YFP expression directly under fluorescent microscopy without using antibodies or fixing the tissues. This will provide further opportunities to study the real time dynamics of the endogenous proteins in living tissues in the future. In conclusion, we have demonstrated the feasibility of these two CPTI lines for future potential applications.

## Supplementary Material


*Ubx^CPTI000601^* and *hth^CPTI000378^* are YFP protein trap insertions in *Ubx* and *hth*, respectively. In the case of *Ubx*, the YFP exon is inserted into the last intron of the gene at genomic position chr3R:12486327. The inserted exon is in the same frame as all six known alternatively spliced transcript variants of *Ubx*. The *hth^CPTI000378^* line is an insertion at genomic position chr3R:6381126 in the endogenous *hth* gene. The insertion traps all but the two shortest *hth* spliced transcript variants (*hth*-RE and *hth*-RF).

## Figures and Tables

**Table 1:** 

Antibody	Raised in	Source/reference	Dilution	Type
*α*-Ubx	Mouse	[8]	1 : 20	Primary
*α*-Hth	Guinea pig	[9]	1 : 200	Primary
Alexa 594 *α*-guinea pig IgG	Goat	Molecular probes	1 : 400	Secondary
Alexa 488 *α*-mouse IgG	Goat	Molecular probes	1 : 400	Secondary
Alexa 488 *α*-rabbit IgG	Goat	Molecular probes	1 : 400	Secondary
*α*-GFP	Rabbit	Molecular probes	1 : 8000	Primary

**Table 2:** The number of Ubx-YFP homozygous and heterozygous flies from crosses between heterozygous *Ubx* ^*CPTI*000601^ flies.

Vial	Total number of counted flies	Observed number of homozygotes	Observed number of heterozygotes
1	197	18	179
2	149	12	137
3	55	3	52
4	51	7	44
5	73	14	59

Total	525	54	471

% Lethality	69.1%		

% Survival	30.9%		

**Table 3:** Chi-square test was performed to check whether the observed numbers (*O*) match with the expected numbers (*E*). The results suggest that the observed numbers are in line with the expected Mendelian ratio. The degrees of freedom (df) are 3 and the probability *P*(*X* ^2^ ≥ 0.37) = 0.9463 on 3 degrees of freedom. This indicates that the difference between the observed data with expected values is not significant. In other words, the number of flies that we observed in each genotype category is in line with what would be expected based on Mendelian ratio.

Genotype	*O*	E	(*O* − *E*)^2^/*E*
*ht* *h* ^*C*1^/TM6C	199	194	0.13
*ht* *h* ^*C*1^/*hth* ^*CPTI*000378^	194	194	0
TM2/TM6C	200	194	0.19
*ht* *h* ^*CPTI*000378^/TM2	191	194	0.05

			0.37

## Abbreviations

GFP: Green fluorescent protein
ChIP: Chromatin immunoprecipitation
YFP: Yellow fluorescent protein
AEL: After egg laying
CNS: Central nervous system.
