# Supplementary material for: Characterisation of Drosophila Ubx CPTI000601 and hth CPTI000378 Protein Trap Lines
Source: ScientificWorldJournal. 2014 Oct 15;2014:191535. doi: 10.1155/2014/191535 (PMC4214163; doi:10.1155/2014/191535)
Supplement: Supplementary file 1 — UbxCPTI000601 and hthCPTI000378 are YFP protein trap insertions in Ubx and hth, respectively. In the case of Ubx, the YFP exon is inserted into the last intron of the gene at genomic position chr3R:12486327. The inserted exon is in the same frame as all six known alternatively spliced transcript variants of Ubx. The hthCPTI000378 line is an insertion at genomic position chr3R:6381126 in the endogenous hth gene. The insertion traps all but the two shortest hth spliced transcript variants (hth-RE and hth-RF). [file 191535.f1.doc]

**Supplementary data**

**
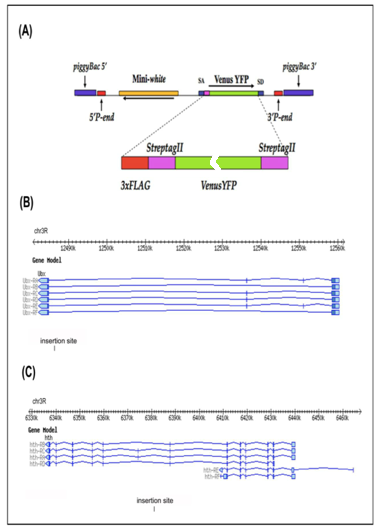
**

Protein traps and insertion sites. (A) A protein trap construct showing the YFP exon flanked by the splicing acceptor (SA) and splicing donor (SD) sites. The construct is randomly introduced into genome using PiggyBac transposition. The expanded region shows the tag sequences incorporated with the VenusYFP. (B) Mapping the CPTI000601 insertion site with respect to *Ubx* transcripts. (C) Mapping the CPTI000378 insertion site with respect to *hth* transcripts.
